# Transforming data to information: A parallel hybrid model for real‐time state estimation in lignocellulosic ethanol fermentation

**DOI:** 10.1002/bit.27586

**Published:** 2020-10-15

**Authors:** Pau Cabaneros Lopez, Isuru A. Udugama, Sune T. Thomsen, Christian Roslander, Helena Junicke, Miguel M. Iglesias, Krist V. Gernaey

**Affiliations:** ^1^ Department of Chemical and Biochemical Engineering, Process and Systems Engineering Center (PROSYS) Technical University of Denmark (DTU) Lyngby Denmark; ^2^ Department of Geosciences and Natural Resource Management University of Copenhagen Frederiksberg Denmark; ^3^ Department of Chemical Engineering Lund University Lund Sweden; ^4^ Department of Chemical Engineering, CRETUS Institute Universidade de Santiago de Compostela Santiago de Compostela Spain

**Keywords:** continuous‐discrete extended Kalman filter, fermentation, hybrid model, lignocellulosic ethanol, spectroscopy

## Abstract

Operating lignocellulosic fermentation processes to produce fuels and chemicals is challenging due to the inherent complexity and variability of the fermentation media. Real‐time monitoring is necessary to compensate for these challenges, but the traditional process monitoring methods fail to deliver actionable information that can be used to implement advanced control strategies. In this study, a hybrid‐modeling approach is presented to monitor cellulose‐to‐ethanol (EtOH) fermentations in real‐time. The hybrid approach uses a continuous‐discrete extended Kalman filter to reconciliate the predictions of a data‐driven model and a kinetic model and to estimate the concentration of glucose (Glu), xylose (Xyl), and EtOH. The data‐driven model is based on partial least squares (PLS) regression and predicts in real‐time the concentration of Glu, Xyl, and EtOH from spectra collected with attenuated total reflectance mid‐infrared spectroscopy. The estimations made by the hybrid approach, the data‐driven models and the internal model were compared in two validation experiments showing that the hybrid model significantly outperformed the PLS and improved the predictions of the internal model. Furthermore, the hybrid model delivered consistent estimates even when disturbances in the measurements occurred, demonstrating the robustness of the method. The consistency of the proposed hybrid model opens the doors towards the implementation of advanced feedback control schemes.

## Graphical abstract

‐ Data‐based models allow measuring key process variables in real‐time. However, they lack predictive power to forecast the progress of the fermentation.
‐ Mechanistic models use the initial conditions to predict the progress of the fermentation, but they cannot account for process deviations.
‐ The hybrid approach uses a continuous‐discrete extended Kalman to incorporate the real‐time measurements obtained with the data‐driven models to the mechanistic models to improve the forecasts.

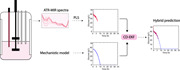

**Abbreviations**
5‐HMF: 5‐hydroxymethyl furfural
ATR‐MIR: attenuated total reflectance mid‐infrared spectroscopy
EtOH: ethanol
FA: furfuryl alcohol
Fur: furfural
Glu: glucose
HAc: acetic acid
HPLC: high‐performance liquid chromatography
PLS: partial least squares
Xyl: xylose

## 1 INTRODUCTION

The transition of fuels and chemicals production from nonrenewable resources to renewables is a key requirement in realizing a circular economy. An example of this transition is the production of ethanol (EtOH; as fuel and chemical platform) from renewable substrates such as lignocellulosic feedstocks, which otherwise would be discarded as waste material (Drapcho et al., 2008; Li et al., 2016). Despite the decades‐long research work on lignocellulosic fermentation, the real‐time monitoring of key state variables in such a fermentation process is required to successfully counter the effect of:

(i) toxicity of the fermentation media derived from lignocellulosic feedstocks,
(ii) the presence of a mixed carbon source in the substrate,
(iii) the inherent variation of the feedstocks, and
(iv) contamination that can occur in industrial settings (Cabaneros et al., 2019; Drapcho et al., 2008).

Fed‐batch process operations, where the feed rate is adjusted to keep the concentration of inhibitors and glucose (Glu) inside the reactor below a certain level, can be useful to mitigate the toxic effects of the inhibitors and to promote the coconsumption of the different carbon sources (Drapcho et al., 2008; Knudsen & Rønnow, 2020; Mauricio‐Iglesias et al., 2015). However, the substrate variability often results in different fermentation profiles between batches, which can result in significant operational challenges. In this context, limiting the feed rate to avoid the effect of the inhibitors, can decrease the productivity of the process, and increasing the length of the fed‐batch process can increase the risk of contamination (Cabaneros et al., 2019; Eliasson Lantz et al., 2010). To optimize the cellulosic EtOH fermentation, it is necessary to develop flexible operations that are able to account for the effects of substrate variability and to react to possible process deviations in real‐time (as the fermentation progresses; Eliasson Lantz et al., 2010). As a consequence, developing and implementing appropriate monitoring schemes to gain real‐time information on the state of the fermentation is crucial to enable control actions and to improve the competitiveness of cellulose‐to‐EtOH processes (Cabaneros et al., 2019; Eliasson Lantz et al., 2010).

With the ever‐increasing intention of biochemical industries to leverage data to improve process operations, there is increased interest in applying measurement methods to monitor processes in real‐time (Udugama et al., 2020) Choosing a suitable monitoring method for cellulose‐to‐EtOH fermentations is challenging due to the high complexity of the fermentation matrix and the high concentration of suspended solids (Cabaneros et al., 2019; Eliasson Lantz et al., 2010). In practice, the commonly monitored variables in fermentation processes, for example, pH, temperature, or pO_2_ often fail at delivering actionable information to design feedback control schemes (Cabaneros et al., 2019). Therefore, it can be clearly seen that more advanced measurements (e.g., of substrates, products, biomass, or inhibitors) are needed to improve the operation of cellulose‐to‐EtOH processes. Different measuring methods are available to monitor the compounds dissolved in the liquid phase (e.g., vibrational spectroscopy, biosensors, or at‐line high‐performance liquid chromatography [HPLC]) or to monitor the biomass concentration (e.g., capacitance probes or fluorescence spectroscopy; Cabaneros et al., 2019). Among the different options available, attenuated total reflectance mid‐infrared spectroscopy (ATR‐MIR) is an analytical tool that allows the fast and simultaneous detection of several compounds (including multiple sugars or weak acids) from the fermentation media (Cabaneros et al., 2019; Lourenço et al., 2012). Unlike other spectroscopic methods, ATR‐MIR spectroscopy measures the light reflected from the sample (instead of the light transmitted through it), making it more robust and suited to monitor systems with a high concentration of suspended solids (Cabaneros et al., 2019; Lourenço et al., 2012). The collected spectra are then analyzed using data‐driven methods, usually, partial least squares (PLS) regressions, to make use of the linear correlations between the concentration of the different analytes and the absorbance in the spectra (Lambert Beer's law; Lourenço et al., 2012). However, the complexity of the media and the highly correlated dynamics between the concentrations of many analytes results in complex spectra with overlapping peaks and require extensive data analysis to train reliable predictive models (Cervera et al., 2009; Krämer & King, 2016). This situation makes the measurements noisy and often unsuited for the implementation of advanced control schemes (Krämer & King, 2017).

Mathematical approaches such as state estimators derived from Kalman filters (KFs; Krämer & King, 2017) or particle filters (Golabgir & Herwig, 2016) have been successfully implemented to address these types of challenges (Golabgir & Herwig, 2016; Krämer & King, 2017; Mauricio‐Iglesias et al., 2015; Price et al., 2014). A continuous‐discrete extended Kalman filter (CD‐EKF) is a particular extension of the KF to nonlinear continuous systems with discrete measurements. This makes the CD‐EKF an appropriate tool to monitor bioprocesses given the nonlinear kinetics of biological systems (Mauricio‐Iglesias et al., 2015; Price et al., 2014; Ricardo, 2019). Similar to the KF, the CD‐EKF algorithm operates iteratively in two steps: a prediction and an update step.

In this study, a hybrid monitoring approach based on CD‐EKF is proposed to estimate the concentration of Glu, xylose (Xyl), and EtOH from spectroscopic measurements collected with ATR‐MIR spectroscopy and to monitor the progression of cellulose‐to‐EtOH fermentations in real‐time (von Stosch et al., 2014). The CD‐EKF reconciliates the predictions made by the internal model (a kinetic model for cellulosic EtOH fermentation) with the PLS predictions of the concentrations of Glu, Xyl, and EtOH. Due to the high complexity and limited availability of fermentation media, the calibration set for the PLS models solely contained synthetic samples that were purposely planned using a design of experiments approach, and no fermentation samples were included in it. This calibration set was carried out to minimize the correlation between the concentration of Glu, Xyl, and EtOH and to distribute the leverage of each sample evenly in the design space.

This developed hybrid approach provides a more stable and robust monitoring framework as it eliminates the deficiencies of a purely mechanistic or data‐driven approach. That is, unmeasured process disturbances and inherent variations in biological systems can lead to significant mismatches with the kinetic model, and data‐driven sensors can be noisy, difficult to interpret, and often lack extrapolation power because they ignore the dynamics of the system. The developed approach was then applied to monitor different cellulose‐to‐EtOH fermentations carried out at the bench scale, and the results obtained were compared to a scenario where only measurements are used to monitor the process.

## 2 MATERIALS AND METHODS

### 2.1 Cell culture growth and propagation

#### 2.1.1 Growth on agar plates

One milliliter of the glycerol stock of the Xyl consuming *Saccharomyces cerevisiae* CEN. PK. XXX (*S. cerevisiae* CEN. PK 122. MDS with overexpression of the native genes *RPE1, TAL1*, and *XKS1*, and with the insertion in the genome of the genes *XYL1* and *XYL2* from *Scheffersomyces stipitis*; Westman et al., 2014) was inoculated in a 250 ml shake flask with 100 ml of yeast extract‐peptone–dextrose medium, containing 10 g/L of yeast extract (Microbiology Fermtech), 20 g/L of peptone from casein, (Microbiology Fermtech), and 20 g/L of dextrose (Sigma‐Aldrich). The shake flask was cultured for 24 h at 30°C and 180 rpm. One milliliter of the grown cell culture was transferred to a 250 ml shake flask containing 100 ml of YPX medium (10 g/L of yeast extract, 20 g/L of peptone, and 20 g/L of Xyl (Sigma‐Aldrich) and it was grown for 36 h at 30°C and 180 rpm. One milliliter of the cell culture grown in YPX was diluted 1000 times, plated in a YPX‐agar plate (YPX media with 10 g/L agar), and incubated for 36 h at 37°C before storage at 4°C.

#### 2.1.2 Cell culture propagation

A single colony of *S. cerevisiae* CEN. PK. XXX grown on YPX‐agar plates was transferred to a 250 ml shake flask containing 100 ml of YPX media, and it was grown for 36 h at 30°C and 180 rpm. One milliliter of the grown cell culture was inoculated in a 500 ml shake flask, filled with 250 ml of YPX media, and grown for 36 h at 30°C and 180 rpm before inoculation of the fermenter. Before inoculating, the dry weight of the cell culture was measured as described in (El‐Mansi et al., 2012).

### 2.2 Fermentation experiments

Four batch fermentations were carried out in a 2.5 L BIOSTAT® A bioreactor (Sartorius) with a working volume of 1.5 L (Table 1), equipped with two 6‐bladed Rushton impellers, pH and temperature control. In all fermentations, the pH was controlled at 6 using 5 M H_2_SO_4_ and 2 M NaOH. The temperature was kept at 30°C and the stirring rate at 450 rpm. The wheat straw hydrolysate was supplemented with 5 g/L of yeast extract and 10 g/L of peptone and centrifuged (Heraeuse Multifuge X3R; Thermo Fisher Scientific) for 10 min at 4000 rpm to reduce the concentration of suspended solid compounds to 10 g/L. In fermentations 2, 3, and 4, the media was centrifuged for another 10 min at 4000 rpm to further reduce the concentration of suspended solid compounds to 2 g/L. A volume of 1.4 L wheat straw hydrolysate (prepared as explained in the Supporting Information Material) was inoculated with 100 ml of grown cell culture. The fermentation lasted between 25 and 35 h until the Xyl was consumed. A sample of 1.5 ml was taken hourly, filtrated through a 0.20 µm cellulose acetate filter (Labsolute USA, 7699822) and stored at −20°C for off‐line analysis with HPLC.

**Table 1:** Overview of the four cellulose‐to‐ethanol batch fermentations

	Fermentation 1	Fermentation 2	Fermentation 3	Fermentation 4
Solid compounds (g/L)	10	2	2	2
Inoculum size (g/L)	1.4	1	0.4	1.4
Initial (glucose) (g/L)	37	37	42	39
Initial (xylose) (g/L)	22	22	22	22
Kinetic model	Identification	Validation	Validation	Validation
Data‐driven model	Not used	Validation	Validation	Validation
Kalman filter	Not used	Tuning	Validation	Validation

### 2.3 Off‐line analysis with HPLC

Glu, Xyl, EtOH, furfural (Fur), 5‐hydroxymethyl furfural (5‐HMF), and acetic acid (HAc) were measured off‐line using an Ultimate 3000 HPLC (Thermo Fisher Scientific) with an Aminex HPX‐87 H column (Bio‐Rad) at 50°C with 5 mM H_2_SO_4_ as eluent and a flow rate of 0.6 ml/min for 80 min. A sample volume of 950 µl was diluted with 50 µl of 5 M H_2_SO_4_ before injection. All compounds were detected using the refractive index (ERC RefractoMax 520; Prague) at 50°C.

### 2.4 Spectroscopy and data collection

**Figure 1:**
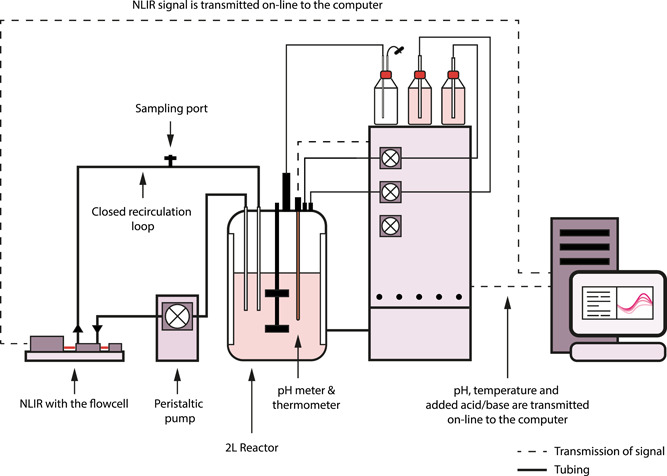
Schematic representation of the set up used for the fermentation [Color figure can be viewed at wileyonlinelibrary.com]

The ATR‐MIR spectrophotometer provided by CellView IVS and manufactured by NLIR APS was connected to the fermenter using a flow‐cell equipped with the ATR crystal (Pike Technologies) and a closed recirculation loop. The media was directed to the flow‐cell from the fermenter and sent back at a flow rate of 90 ml/min. To ensure that the readings in the flow cell reflect the dynamics in the fermenter, the cells must spend as little time as possible in the recirculation loop. For this reason, the length of the tubing in the closed‐loop was chosen to limit the residence time in the tubing to 20–25 s. Background and reference measurements were taken before starting the fermentation by measuring the spectrum of air first with the laser turned off and then with the laser turned on. The exposure time of the sample was set to 120 ms. Every minute, 100 ATR spectra of the fermentation media were taken and their average was recorded and stored as a single. txt file. Each spectrum contained absorbance data in the range of 428–1833 cm^−1^ with a resolution of 1 cm^−1^. A schematic representation of the experimental set‐up is given in Figure 1.

## 3 STATE ESTIMATION

### 3.1 Hybrid monitoring algorithm for real‐time state estimation

**Figure 2:**
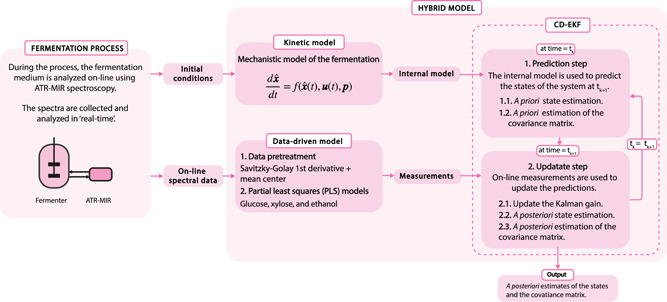
Scheme of the hybrid monitoring approach [Color figure can be viewed at wileyonlinelibrary.com]

The algorithm proposed for on‐line monitoring uses a CD‐EKF to make estimations of the state variables. The CD‐EKF fuses the predictions made with the kinetic model and the measurements calculated with the data‐driven model to give an estimate of the real system state (Figure 2). The kinetic model is a time‐continuous system of differential equations containing the reaction rates and the stoichiometry of the different state variables of the fermentation (with the form shown in Equation 1)

(1) $$\frac{d\hat{x}}{\mathrm{dt}}=f(\hat{x}\left(t\right),u\left(t\right),p),$$

where $\hat{x}\left(t\right)$ is a vector containing the state variables of the model, $u\left(t\right)$ are the external inputs, and p are the model parameters. The data‐driven model consists of a set of three independent PLS models that take the spectra collected on‐line with the ATR‐MIR spectrometer as input and return the measured concentrations of Glu, Xyl, and EtOH as output.

At time zero ($t_{0}$), the mechanistic model is used to generate a long‐horizon prediction of the profile of the fermentation from the initial conditions (${\hat{x}}_{0}$). Then, during the fermentation, the CD‐EKF operates iteratively in two steps to predict and update the estimations of the concentrations of Glu, Xyl, and EtOH. In the first step, at each time $t_{k}$, given the previous *a posteriori* estimate of the state of the system ${\hat{x}}_{kk}$, the kinetic model is integrated numerically from $t_{k}$ to $t_{k+1}$ to produce a priori estimates of the states at $t_{k+1}$ (${\hat{x}}_{k+1k}$).

(2) $${\hat{x}}_{k+1k}={\hat{x}}_{kk}+\int_{t_{k}}^{t_{k+1}}f(\hat{x}\left(t\right),u\left(t\right),p)\mathrm{dt}+w_{k},$$

where $w_{k}\sim N(0,Q)$ represents the process noise (assumed to be independent and normally distributed). During the same step, a priori estimates of the covariance matrix (${P^{x}}_{k+1k}$) are also calculated by propagating it forward from $t_{k}$ to $t_{k+1}$ using Equation (3) and taking the ${P^{x}}_{kk}$ as initial conditions (Zhou et al., 2012).

(3) $$P_{k+1|k}=A\left(t\right)P_{k|k}{A(t)}^{T}+Q,$$

where $A\left(t\right)$ is the Jacobian matrix of the kinetic model in the time interval from $t_{k}$ to $t_{k+1}$. At $t_{k+1}$ (every 15 min), 15 new spectra have been collected (every minute from $t_{k}$ to $t_{k+1}$), and the PLS models calculate the concentration of Glu, Xyl, and EtOH. Then the median values of the 15 predicted concentrations of Glu, Xyl, and EtOH are used to produce the measurement vector ${\hat{y}}_{k+1}$. The measurement vector ${\hat{y}}_{k+1}$ relates to the state of the system at $t_{k+1}$ following equation:

(4) $${\hat{y}}_{k+1}=h({\hat{x}}_{k+1})+v_{k+1},$$

where the function $h({\hat{x}}_{k+1})$ relates the states at $t_{k+1}$ to the measurement vector ${\hat{y}}_{k+1}$ and $v_{k+1}\sim N(0,R)$ is the noise associated with the measurements. At $t_{k+1}$, the new measurements of the concentration of Glu, Xyl, and EtOH are used to update the a priori estimates of the state variables and their covariance matrix ${(\hat{x}}_{k+1k},{P^{x}}_{k+1k})$ to produce the *a posteriori* estimates ${(\hat{x}}_{k+1k+1},{P^{x}}_{k+1k+1})$. This is the update step in the CD‐EKF algorithm. First, the Kalman gain ($K_{k+1}$) is calculated using Equation (5) where C is the Jacobian matrix of $h({\hat{x}}_{k+1})$. Finally, a posteriori estimates of the states and the covariance matrix are calculated using the update equations (Equations 6 and 7, respectively).

(5) $$K_{k+1}={P^{x}}_{k+1k}C^{T}{\left(C{P^{x}}_{k+1k}C^{T}+R\right)}^{-1},$$

(6) $${\hat{x}}_{k+1k+1}={\hat{x}}_{k+1k}+K_{k+1}\left({\hat{y}}_{k+1}-C{\hat{x}}_{k+1k}\right),$$

(7) $${P^{x}}_{k+1k+1}=(I-K_{k+1}C){P^{x}}_{k+1k},$$

where I is the identity matrix. At the end of each iteration, the *a posteriori* covariance matrix ${{(P}^{x}}_{k+1k+1})$ is used to calculate the 95% confidence interval of the *a posteriori* state estimates

(8) $${\hat{x}}_{k+1k+1}={\hat{x}}_{k+1k+1}\pm 3\sigma_{k+1},$$

where $\sigma_{k+1}^{2}=\text{diag}({P^{x}}_{k+1k+1})$.

Before building the hybrid model, it is necessary to develop, identify, and calibrate the kinetic and the PLS models. The kinetic model was identified off‐line using data from fermentation 1 (Table 1), and the PLS models were calibrated using semi‐synthetic samples.

### 3.2 Kinetic model

**Figure 3:**
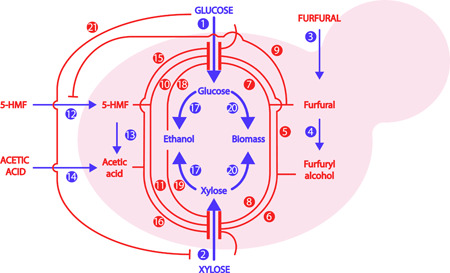
Conceptualization of the kinetic model. (1) Glucose uptake, (2) xylose uptake, (3) furfural uptake, (4) furfural is converted into furfuryl alcohol (FA), (5) FA inhibits the uptake of glucose, (6) FA inhibits the uptake of xylose, (7) furfural inhibits the uptake of glucose, (8) furfural inhibits the uptake of xylose, (9) furfural inhibits the uptake of 5‐hydroxymethyl furfural (5‐HMF), (10) 5‐HMF inhibits the uptake of glucose, (11) 5‐HMF inhibits the uptake of xylose, (12) 5‐HMF uptake, (13) 5‐HMF is converted into acetate, (14) acetic acid uptake, (15) acetic acid inhibits the uptake of glucose, (16) acetic acid inhibits the uptake of xylose, (17) production of ethanol, (18) ethanol inhibits the uptake of glucose, (19) ethanol inhibits the uptake of xylose, (20) cell growth, (21) competitive inhibition: glucose inhibits the uptake of xylose [Color figure can be viewed at wileyonlinelibrary.com]

The kinetic model describing the dynamics of Glu, Xyl, Fur, furfuryl alcohol (FA), 5‐HMF, HAc, EtOH, and biomass was implemented in Matlab 2016® (Mathworks). The model, developed by Mauricio‐Iglesias et al. (2015), describes the growth of *S. cerevisiae* on Glu and Xyl and accounts for the inhibitory effects of Fur, FA, 5‐HMF, HAc, and EtOH. A graphical conceptualization of the different processes in the model is shown in Figure 3.

The kinetic model was developed following the notation described by Sin et al. (2008). The model was based on the uptake rates of Glu, Xyl Fur, 5‐HMF, and HAc and the reaction rates of FA, EtOH, and biomass were expressed as a linear combination of the uptake rates of Fur, Glu, and Xyl using the stoichiometric matrix (shown in Table 2). The reaction rates for each state variable were modeled using the following expressions:

- The uptake rate of the substrates (Glu and Xyl) followed Monod kinetics with substrate inhibition (Equation 9 in Table 3).
- The uptake rate of the inhibitors (Fur, 5‐HMF, and HAc) followed regular Monod kinetics (Equation 10 in Table 3).
- The inhibition effects by Fur, FA, 5‐HMF, and HAc were modeled by multiplying the reaction rates by the inhibition term shown in Equation 11 in Table 3.
- Product inhibition of the uptake rates of Glu and Xyl was modeled by multiplying the respective uptake rates by the empirical term shown in Equation 12 in Table 3.
- Competitive inhibition (Glu inhibits the uptake of Xyl) was modeled using the inhibitory term shown in Equation 13 in Table 3.

**Table 2:** Stoichiometric matrix

	Gluc	Xyl	Fur	FA	5‐HMF	HAc	EtOH	X
Glu uptake	−1	0	0	0	0	0	*Y*_EtOH/Glu_	*Y*_X/Glu_
Xyl uptake	0	−1	0	0	0	0	*Y*_EtOH/Xyl_	*Y*_X/Xyl_
Furuptake	0	0	−1	*Y*_FA/Fur_	0	0	0	0
5‐HMF uptake	0	0	0	0	−1	*Y*_HAc/5‐HMF_	0	0
HAc uptake	0	0	0	0	0	−1	0	0

Abbreviations: 5‐HMF, 5‐hydroxymethyl furfural; X, biomass; EtOH, ethanol; FA, furfuryl alcohol; Fur, furfural; Glu, glucose; HAc, acetic acid; Xyl, xylose.

**Table 3:** Mathematical terms describing the reaction rates of the model

Compound	Reaction rate	Compounds	Equation no.
**Substrate uptake**	$\frac{v_{\max,S}∙S}{K_{S}+S+\frac{S^{2}}{K_{i,S}}}$	S = glucose, xylose	**9**
**Inhibitors uptake**	$\frac{v_{\max,I}∙I}{K_{\mathrm{SP},I}+I}$	I = furfural; 5‐HMF, acetic acid	**10**
**Inhibition effects**	$\frac{1}{1+\frac{I}{K_{i,I,S}}}$	S = glucose, xylose; I = furfural, furfuryl alcohol; 5‐HMF, acetic acid	**11**
**Product inhibition**	$1-{\left(\frac{P}{{P_{\max,S}}^{†}}\right)}^{\gamma_{S}}$	S = glucose, xylose; P = ethanol	**12**
**Competitive inhibition**	$\frac{1}{1+\frac{I}{K_{i,I,S}}}$	S = xylose; I = glucose	**13**

Abbreviation: 5‐HMF, 5‐hydroxymethyl furfural.

^†^ $P_{\max,S}$ is the product inhibition constant.

By combining the stoichiometric matrix with the uptake kinetic rates, a model with 8 ordinary differential equations and 32 parameters was obtained (the full set of differential equations and the list of parameters are shown in the Supporting Information Material). The parameters were estimated by fitting the model to off‐line experimental data obtained with HPLC. The profiles of Glu, Xyl, Fur, and EtOH of fermentation 1 (Table 1) were used for the parameter estimation (the results of the parameter estimation are shown in Supporting Information Material). In brief, first parameter estimation was performed using the nonlinear least‐squares method to fit the maximum biomass specific uptake rates of Glu, Xyl, and HAc to the experimental data (*v*_max, Glu_, *v*_max, Xyl_, *v*_max, HAc_) using the *lqnonlin* function of Matlab Release 2016b. The initial conditions are shown in Table 1. The parameters found by Krishnan et al. (1999) and Hanly and Henson (2014) were used as the initial guess for the parameter estimation (see Supporting Information Material). To improve the fitting, a local sensitivity and identifiability analysis was conducted to find the parameters with a larger impact on the output, and the combinations of parameters that are not linearly correlated (Brun & Reichert, 2001). As a result of the sensitivity and identifiability analysis, a subset of three parameters (*v*_max, Glu_, *K*_iHAc, Glu_, and *K*_i, Glu, Xyl_) was selected for further estimation (the results of the sensitivity and identifiability analyses are shown in Supporting Information Material). A second parameter estimation was performed with the three selected parameters using the bootstrap framework described by Sin and Gernaey (2016). First, a reference parameter estimation was done using nonlinear least squares. Then, 100 synthetic data sets were created by randomly sampling from the residuals of the reference parameter estimation using the Monte Carlo method (Sin & Gernaey, 2016). The three parameters were then re‐estimated for each of the 100 synthetic data sets using nonlinear least squares. This process resulted in a population of 100 estimates for each parameter. The mean, the *SD*, and the covariance matrix were calculated for each of the estimated parameters to assess their uncertainty. The uncertainty in the estimated parameters was propagated through the model using a Monte Carlo approach to assess the uncertainty in the model output (Sin & Gernaey, 2016). The uncertainty in the parameters estimated using the bootstrapping method was considered to follow a normal distribution. The model was finally validated with the fermentations 2–4 (validation results are shown in Supporting Information Material IV).

### 3.3 Calibration of the data‐driven models

**Figure 4:**
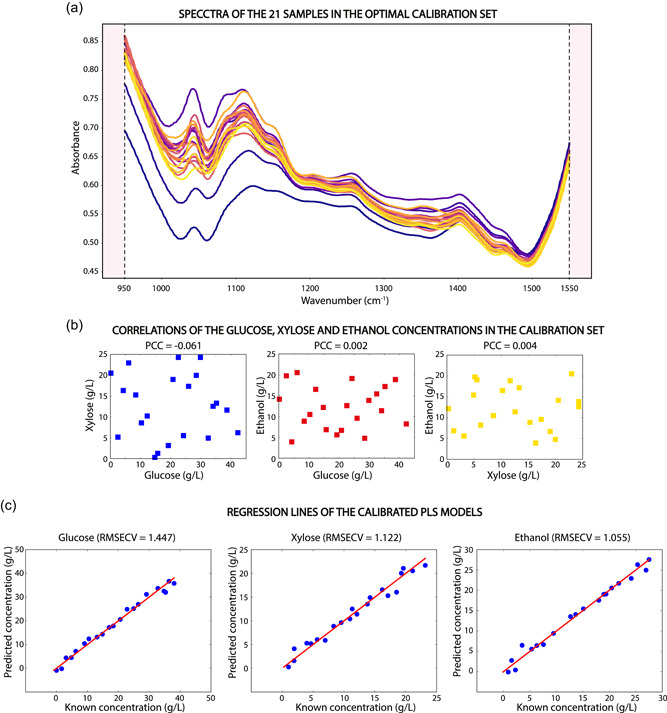
(a) Spectra of the calibration samples in the optimal calibration set obtained from Latin hypercube sampling. (b) Pairwise Pearson's correlation (PCC) between the concentration of glucose, xylose, and ethanol in the calibration set. (c) Root mean squared error during the cross‐validation (RMSECV) of the partial least squares (PLS) models for glucose, xylose, and ethanol [Color figure can be viewed at wileyonlinelibrary.com]

Three independent PLS models were developed to calculate the concentrations of Glu, Xyl, and EtOH from the spectral data collected with the ATR‐MIR spectrophotometer. Due to the limited medium availability, a specific procedure to calibrate the PLS models was designed to (1) account for the matrix absorbance; (2) distribute the leverage of each calibration sample evenly along the experimental space; and (3) minimize the correlation between the concentrations of Glu, Xyl, and EtOH. First, 1.5 L of wheat straw hydrolysate was fermented (as described in Section 2) to remove the Glu and Xyl from the media (the Glu was entirely removed, but a residual concentration of 0.2 g/L of Xyl remained in the fermentation media). Then, the fermented medium was centrifuged (at 4000 rpm for 5 min) to remove the biomass, and the EtOH was stripped out by sparging sterile air for 24 h at 35°C. Finally, the volume was adjusted to 1.5 L by adding 150 ml of deionized water. The resulting broth was the fermentation matrix without Glu, Xyl, or EtOH, and it was used to prepare 21 semi‐synthetic samples for the calibration set. Note that the fermentation matrix used to calibrate the PLS models corresponded to the matrix at the end of the fermentation, and therefore, the PLS models did not take into account the changes in the fermentation matrix. This approach is valid under the assumption that the contribution to the variance of the spectral matrix caused by the change in the concentration of the analytes is much more significant than the contribution caused by the change in the matrix. The samples were prepared based on a three dimensional Latin hypercube (LH) experimental design. This design was chosen to distribute the leverage of the different samples evenly along with the experimental space (Montgomery, 2009; the calibration space is shown in Table 4). To minimize the correlation between the concentrations of Glu, Xyl, or EtOH in the different samples, 100,000 randomized LH candidates were created and ranked according to their average pairwise Pearson's correlation coefficients (PCCs). The calibration design with the lowest correlation was selected. The spectra corresponding to the samples in the selected calibration set, as well as the pairwise PCC, are shown in Figure 4 (the specific concentrations are shown in the Supporting Information Material). This allows the PLS to provide independent results in fermentations with different dynamics.

**Table 4:** Calibration space considered for the partial least squares models

	Lower limit	Upper limit
Glucose (g/L)	0	40
Xylose (g/L)	0	25
Ethanol (g/L)	2.5	32.5

A volume of 75 ml was prepared for each sample, and the specific concentration of Glu, Xyl, and EtOH was determined by HPLC. The spectrum of each sample was collected by circulating it through the flow cell. The spectral area of the calibration set between 950 and 1550 cm^−1^ was used to calibrate three independent PLS models for Glu, Xyl, and EtOH, respectively, using the MBPLS package in Python 3.7 (Baum & Vermue, 2019). The spectra were preprocessed by taking the first derivative and mean centering them. A leave‐one‐out cross‐validation procedure was used to calculate the optimal number of latent variables (LV) for each model by minimizing the root mean square error of cross‐validation (RMSECV). The PLS models for Glu, Xyl, and EtOH consisted of 4, 3, and 3 LVs, respectively (Supporting Information Materials). In all cases, the PLS models described more than 95% of the variance in both, the *X* (spectra) and *Y* (concentration) blocks. The regression lines of each PLS model are shown in Figure 4c.

### 3.4 Tuning the CD‐EKF

To initialize the CD‐EKF, initial estimates of the measurement and process noise covariance matrices must be provided. This step is crucial to determine the degree of reliance of the *a posteriori* estimates on the a priori estimates and on the measurements. The measurement noise covariance matrix (R) was found directly from the variance of residuals between the HPLC measurements and the PLS predictions. During the Glu consumption phase, the production of other compounds (e.g., glycerol) interfered with the PLS predictions of Xyl and EtOH, resulting in much lower measurement accuracy (i.e., the measurement noise covariance of Xyl and EtOH was nearly twice as high during the Glu consumption phase than during the rest of the fermentation). To account for this different prediction quality, two measurement noise variance matrices were used for the Glu and Xyl consumption phases ($R_{1}$ and $R_{2}$, respectively; Ricardo, 2019). Since Glu inhibits the consumption of Xyl, it was set that the Xyl consuming phase started when the Glu concentration was below ¼ of the value of the inhibition constant of Glu on Xyl ($K_{i,\mathrm{Glu},\mathrm{Xyl}}$). Before this point, $R_{1}$ was used as the variance matrix of the measurements, and $R_{2}$ was used afterward. The initial process noise covariance matrix (Q) was iteratively tuned following the procedure described by Price et al. (2014). First, a small Q $\left(1\times 10^{-7}\right)$ was chosen, which made the CD‐EKF rely excessively on the internal model and made it insensitive to the measurements. Then, Q was gradually increased until the estimated states matched the off‐line measurements taken with HPLC (Q $=1\times 10^{-5})$. The tuning of the CD‐EKF was done in fermentation 2, and fermentations 3 and 4 were used to validate the monitoring approach using different fermentation conditions (Table 1).

## 4 RESULTS AND DISCUSSION

**Figure 5:**
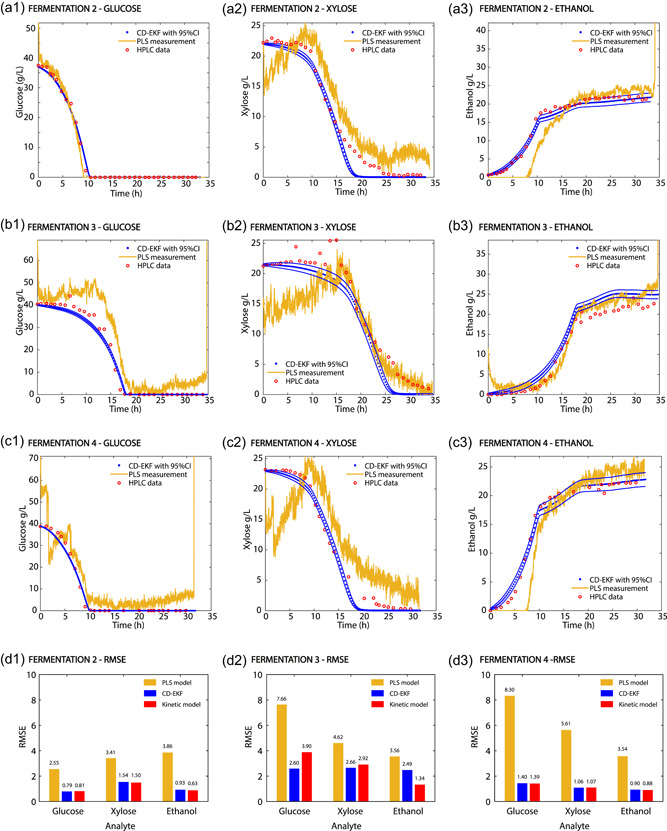
(a–c) Comparison between the monitoring outcomes of the partial least squares (PLS) and the hybrid model in fermentations 2–4. Fermentations 2–4 were validation sets of the PLS model, while the continuous‐discrete extended Kalman filter (CD‐EKF) was tuned using fermentation 2 and validated in fermentations 3–4. (d1–3.) The root‐mean‐squared error (RMSE) between the hybrid model, the PLS, the kinetic model, and the off‐line high liquid performance chromatography (HPLC) samples [Color figure can be viewed at wileyonlinelibrary.com]

The performances of two real‐time monitoring strategies were compared using three different cellulose‐to‐EtOH fermentations. The first scenario only relied on the data‐driven model to predict the concentrations of Glu, Xyl, and EtOH. The second scenario used the hybrid model to reconciliate the measurements from the data‐driven model with the internal model of the process to estimate the system states. The performance of each method was compared using the RMSE between the off‐line measurements taken with HPLC and the real‐time predictions (Figure 5).

### 4.1 Strategy 1: Monitoring using a data‐driven model only

The PLS predictions of the concentrations of Glu, Xyl, and EtOH for the fermentations 2–4 are shown in Figure 5a–c. Since the calibration set of the PLS models only included synthetic samples generated using the LH sampling and did not include any fermentation samples, the results are shown in Figure 5a–c are three independent validations of the models. The PLS models were able to describe the profiles of Glu, Xyl, and EtOH in all three batch fermentations with different accuracies (Figure 5). The prediction of Glu was accurate in fermentation 2 (Figure 5a1), but showed some deviations in fermentations 3 and 4, especially towards the end of the fermentation (Figures 5b1 and 5c1). Note that clogging of the circulation loop occurred after 2 h in fermentation 4 due to the accumulation of suspended solids and caused the dramatic drop in the predicted Glu concentration seen in fermentation 4 (Figure 5c1). The prediction of Xyl followed two well‐distinguished trends during the three fermentations (Figures 5a2, 5b2, and 5c2). The first one occurred during the Glu consumption phase, where the predicted Xyl concentration increased from 15 to 25 g/L. This tendency was neither in accordance with the off‐line measurements nor with the biology of the system (i.e., *S. cerevisiae* does not produce Xyl) and suggested that other factors, neglected in the calibration set of the PLS models, interfered with the prediction of Xyl. This interference was arguably due to the accumulation of glycerol and biomass in the fermentation media during the growth on Glu. *S. cerevisiae* produces glycerol to regenerate NAD^+^/NADH and to maintain the redox balance within the cells (Palmqvist et al., 1999). This was further confirmed by the off‐line measurements with HPLC, which showed that during the Glu consumption phase, glycerol reached a concentration of 3 g/L (data not shown). Moreover, during the Xyl consuming phase, the glycerol concentration did not significantly change, and glycerol remained in the fermentation matrix after stripping the EtOH. In consequence, the matrix used to calibrate the PLS models contained a high‐glycerol concentration and a low‐biomass concentration, which did not represent the properties of the matrix at the beginning of the fermentation. The second trend in the prediction of Xyl occurred during the Xyl consumption phase, where the glycerol concentration remained constant. In this second phase, the PLS model was able to describe the trend of Xyl in all the fermentation, with a slight overestimation in fermentations 2 and 4 (Figures 5a2, 5b2, and 5c2). EtOH was accurately predicted in fermentation 3 (Figure 5b3), but it showed significant deviations during the Glu consumption phase in fermentations 2 and 4 (Figures 5a3 and 5c3). Likely, these deviations are also caused by matrix effects. Nonetheless, during the Xyl consumption phase, the concentration of EtOH is accurately predicted in all fermentations (Figure 5a3, 5b3, and 5c3). The PLS models were able to successfully describe the general trajectories of Glu, Xyl, and EtOH in three fermentations with different initial conditions. These predictions can be used to assess the progress of the fermentation and demonstrate the robustness of the calibration procedure followed to calibrate the models. However, the matrix effects had a substantial interference with the predictions of the PLS models (especially for Xyl and EtOH), making the measurements noisy and unsuited to apply advanced control strategies. The PLS predictions could be improved by expanding the calibration set by including either fermentation samples (to better account for the changes of the fermentation matrix) or by considering glycerol and biomass in the experimental space. Unfortunately, this was not possible due to the limited availability of fermentation media.

### 4.2 Strategy 2: Hybrid monitoring approach

The initial process noise covariance matrix (Q) of the CD‐EKF was tuned in fermentation 2 by iteratively adjusting it to match the CD‐EKF estimations with the off‐line HPLC measurements. After tuning the CD‐EKF, its predictions were validated using fermentations 3 and 4. The performance of the hybrid model was investigated by calculating the RMSE of the estimated states at the different measurement points and by comparing it to the RMSE of the predictions made with the data‐driven model and the internal model. The results showed that in all fermentations, the state estimations made with the hybrid model significantly improved the predictions of the data‐driven model (the RMSE of the hybrid model estimates were between 1.3 and 6 times lower than the RMSE of the predictions made with the PLS model). These results indicate that the hybrid model successfully used the internal model to correct the inconsistencies and noise associated with the PLS predictions. The estimated concentrations of Glu, Xyl, and EtOH made with the hybrid model in fermentation 2 were in excellent agreement with the off‐line measurements and with the internal model of the process (the RMSE of the hybrid and the kinetic model estimates were very similar and below 1.55 g/L in all the states). The validation experiments had different initial conditions that resulted in different fermentation profiles (Table 1 and Figure 5b,c). In fermentation 3, the kinetic model was not able to predict the concentrations of Glu and Xyl with the same accuracy as in fermentation 2. While in fermentation 2 the RMSE of the kinetic model for Glu was 0.97 and 1.54 g/L for Xyl, in fermentation 3 the kinetic model predicted Glu and Xyl with an RMSE of 3.90 and 2.92 g/L, respectively. The predictions improved with the hybrid model, resulting in the lowest RMSE of 2.60 and 2.66 g/L for Glu and Xyl, respectively Figure 5d2), when compared to the predictions of the PLS and the kinetic model. This demonstrates that the hybrid approach is sensitive to the measured data and incorporates it to correct the estimates of the system states, making the hybrid model able to account for process deviations. On the other hand, the prediction of EtOH had a higher RMSE than the internal model due to the overestimation of EtOH concentrations towards the end of the fermentation (Figures 5b3 and 5d2). Similar to fermentation 2, the state estimations of the hybrid model and the predictions of the internal model agreed in fermentation 3 and had an RMSE below 1.4 g/L for each compound. Interestingly, the predictions of the hybrid model were not affected by the dramatic drop in the concentration of Glu and Xyl that occurred in fermentation 4 due to the clogging of the recirculation loop (Figures 5c1–3 and 5d3). As such, it can be clearly seen that the proposed hybrid model is robust to punctual deviations in the measuring signal, which the PLS model lacks. This stability is desirable when dealing with spectroscopic data, where the signal is highly sensitive to disturbances such as air bubbles or solid compounds.

### 4.3 Perspectives for industrial application of the hybrid approach

In the current implementation, the hybrid model was updated with new measurements every 15 min to produce new estimates of the system state. This updating frequency allows monitoring of the progress of the fermentation in real‐time, and can be used to detect deviations in the fermentation profile (e.g., due to contamination by lactic acid bacteria) and to take corrective actions. However, given the dynamics of the system, other control applications (such as feed rate control) would require higher updating frequencies. The data acquisition and the computational time to solve the hybrid model are the two factors limiting the updating frequency. One of the main advantages of using spectroscopic methods over other monitoring tools such as at‐line HPLC is that spectroscopy allows the fast and automated collection and analysis of new spectra, resulting in a high updating frequency of the state variables without the need for manual sampling (Cabaneros et al., 2019). The spectrophotometer used in this study can collect a new spectrum every minute, and the hybrid model is solved in a few seconds, updating the states of the system every 1.5 min. The possibility to reach high updating frequencies makes this approach also useful for the implementation of control schemes that require faster response times (e.g., for feed‐rate control in fed‐batch operations).

In the present work, the media was centrifuged before the fermentation to remove suspended solids compounds that could clog the tubing in the recirculation loop. However, in industrial operations, the media is not centrifuged, and high concentrations of suspended solids are present during the fermentation process. This situation would be further aggravated in processes using simultaneous saccharification and fermentation, where the concentration of solid matter is especially high. Such high concentrations of suspended solid compounds can interfere with the collected spectra, reducing the accuracy of the predictions, and even limiting the industrial applicability of some spectroscopic methods (Cabaneros et al., 2019). In this context, ATR‐MIR spectroscopy is an attractive option due to its robustness to high concentrations of suspended solids caused by the shallow penetration depth of the light into the media. As such, ATR‐MIR spectroscopy has been successfully applied to monitor the progress of various systems with high solid matter on‐line, such as the mashing process in breweries (Patent No. WO 2015/155353, 2015). Although ATR‐MIR spectroscopy can be used to monitor processes with a high concentration of suspended solids, it would still be expected that the interference of the light with the solid matter affects the precision and accuracy of the measurements. On a practical note, even though on‐line proves with a recirculation loop have been successfully implemented in systems with high concentrations of suspended solids, in‐line probes are advantageous to avoid clogs in the recirculation loop (Cabaneros et al., 2019).

## 5 CONCLUSIONS

Real‐time monitoring of cellulose‐to‐EtOH fermentations is challenging due to the high complexity of the fermentation media derived from lignocellulosic material. The results of this study showed that relying only on advanced spectroscopic measurements combined with PLS regression models to measure the concentration of Glu, Xyl, and EtOH can yield a good qualitative description of the fermentation progress. However, the interference of other compounds such as glycerol or biomass and the presence of bubbles and suspended solids in the fermentation broth results in noisy and biased predictions that limit the implementation of advanced control schemes. The hybrid approach presented in this study efficiently fuses the predictions of the PLS model and the internal model of the system to correct the inconsistencies of the PLS predictions and produce consistent estimates of the state variables. Having a thorough understanding of the behavior of the measuring system is crucial to tune a robust and stable CD‐EKF effectively. The hybrid model presented in this study was calibrated and tuned using only two fermentations, and large amounts of data were not required to develop the state estimator. This is an essential feature as data is often not easily available in industrial setups. The quality of the predicted concentrations of Glu, Xyl, and EtOH using the hybrid model opens the doors towards the implementation of advanced monitoring schemes. In the current configuration, the hybrid model can be used to monitor fermentations in real‐time, to detect deviations in the behavior, and to take corrective actions when needed. Moreover, the high sampling frequency (one sample per minute) and the low computational time required by the model allow updating the estimates every 1.5 min, making this approach suitable to implement real‐time control strategies.

## AUTHOR CONTRIBUTIONS

Pau Cabaneros Lopez, Isuru A. Udugama, and Miguel Mauricio Iglesias conceived the ideas and designed the experiments and analyzed the data. Pau Cabaneros Lopez, Sune Tjalfe Thomsen, and Christian Roslander performed the experiments. Pau Cabaneros Lopez and Miguel Mauricio Iglesias implemented the mathematical framework of the hybrid model. Pau Cabaneros Lopez wrote the manuscript and all the authors contributed revising the final manuscript.

## Supporting information

Supporting information.

## Data Availability

Data available on request from the authors.

## References

[bit27586-bib-0001] Baum, A. , & Vermue, L. (2019). Multiblock PLS: Block dependent prediction modeling for Python. Journal of Open Source Software, 4(34), 1190 10.21105/joss.01190

[bit27586-bib-0002] Brun, R. , & Reichert, P. (2001). Practical identifiability analysis of large environmental simulation. Water Resources Research, 37(4), 1015–1030. 10.1029/2000WR900350

[bit27586-bib-0003] Cabaneros, P. , Feldman, H. , Mauricio‐iglesias, M. , Junicke, H. , Kjøbsted, J. , & Gernaey, K. V. (2019). Benchmarking real‐time monitoring strategies for ethanol production from lignocellulosic biomass. Biomass and Bioenergy, 127, 105296 10.1016/j.biombioe.2019.105296

[bit27586-bib-0004] Cervera, A. E. , Petersen, N. , Lantz, A. E. , Larsen, A. , & Gernaey, K. V. (2009). Application of near‐infrared spectroscopy for monitoring and control of cell culture and fermentation. Biotechnology Progress, 25(6), 1561–1581. 10.1002/btpr.280 19787698

[bit27586-bib-0005] Drapcho, C. M. , Nhuan, N. P. , & Walker, T. H. (2008). Biofuels engineering process technology. Mc Graw‐Hill Education 10.1036/0071487492

[bit27586-bib-0006] Eliasson Lantz, A. , Gernaey, K. V. , Franzén, C. J. , & Olsson, L. (2010). On‐line monitoring of fermentation processes in lignocellulose‐to‐bioalcohol production In K. Waldron (Ed.), Biochemical conversion of lignocellulosic biomass (pp. 315–339). Woodhead Publishing Ltd.

[bit27586-bib-0007] El‐Mansi, E. , Bryce, C. , Hartley, B. , & Demain, A. (2012). Fermentation microbiology and biotechnology (3rd ed., pp. 47–98). Taylor & Francis 10.1201/b11490-2

[bit27586-bib-0008] Golabgir, A. , & Herwig, C. (2016). Combining mechanistic modeling and Raman spectroscopy for real‐time monitoring of fed‐batch Penicillin production. Chemie‐Ingenieur‐Technik, 88(6), 764–776. 10.1002/cite.201500101

[bit27586-bib-0009] Hanly, T. J. , & Henson, M. A. (2014). Dynamic model‐based analysis of furfural and HMF detoxification by pure and mixed batch cultures of *S. cerevisiae* and *S. stipitis* . Biotechnology and Bioengineering, 111(2), 272–284. 10.1002/bit.25101 23983023

[bit27586-bib-0011] Knudsen, J. D. , & Rønnow, B. (2020). Extended fed‐batch fermentation of a C5/C6 optimised yeast strain on wheat straw hydrolysate using an online refractive index sensor to measure the relative fermentation rate. Nature Scientific Reports, 10, 6705 10.1038/s41598-020-63626-z PMC7174321 32317712

[bit27586-bib-0012] Krishnan, M. S. , Ho, N. W. Y. , & Tsao, G. T. (1999). Fermentation kinetics of ethanol production from glucose and xylose by recombinant *Saccharomyces* 1400(pLNH33). Applied Biochemistry and Biotechnology ‐ Part A Enzyme Engineering and Biotechnology, 77–79, 373–388. 10.1385/abab:78:1-3:373 15304708

[bit27586-bib-0013] Krämer, D. , & King, R. (2016). On‐line monitoring of substrates and biomass using near‐infrared spectroscopy and model‐based state estimation for enzyme production by *S. cerevisiae* . IFAC‐PapersOnLine, 49(7), 609–614. 10.1016/j.ifacol.2016.07.235

[bit27586-bib-0014] Krämer, D. , & King, R. (2017). A hybrid approach for bioprocess state estimation using NIR spectroscopy and a sigma‐point Kalman filter. Journal of Process Control, 82, 91–104. 10.1016/j.jprocont.2017.11.008

[bit27586-bib-0015] Li, C. , Aston, J. E. , Lacey, J. A. , Thompson, V. S. , & Thompson, D. N. (2016). Impact of feedstock quality and variation on biochemical and thermochemical conversion. Renewable and Sustainable Energy Reviews, 65, 525–536. 10.1016/j.rser.2016.06.063

[bit27586-bib-0016] Lourenço, N. D. , Lopes, J.a , Almeida, C. F. , Sarraguça, M. C. , & Pinheiro, H. M. (2012). Bioreactor monitoring with spectroscopy and chemometrics: A review. Analytical and Bioanalytical Chemistry, 404(4), 1211–1237. 10.1007/s00216-012-6073-9 22644146

[bit27586-bib-0017] Mauricio‐Iglesias, M. , Gernaey, K. V. , & Huusom, J. K. (2015). *12th International Symposium on Process Systems Engineering and 25th European Symposium on Computer Aided Process Engineering. Copenhagen, Denmark*, & (). State estimation in fermentation of lignocellulosic ethanol. Focus on the use of pH measurements. 10.1016/B978-0-444-63577-8.50140-6

[bit27586-bib-0018] Montgomery, D. C. (2009). Design and analysis of experiments (8th ed.). John Wiley & Sons.

[bit27586-bib-0019] Palmqvist, E. , Almeida, J. S. , & Hahn‐Hägerdal, B. (1999). Influence of furfural on anaerobic glycolytic kinetics of *Saccharomyces cerevisiae* in batch culture. Biotechnology and Bioengineering, 62(4), 447–454. 10.1002/(SICI)1097-0290(19990220)62:4<447::AID-BIT7>3.0.CO;2-0 9921153

[bit27586-bib-0020] Price, J. , Nordblad, M. , Woodley, J. M. , & Huusom, J. K. (2014). Real‐time model based process monitoring of enzymatic biodiesel production. Biotechnology Progress, 30(6), 1247–1501. 10.1002/btpr.2030 25504750

[bit27586-bib-0021] Ricardo, C. (2019). *Model‐based monitoring and optimization of a bio‐based process* (Ph.D. thesis, Technical University of Denmark (DTU)). Retrieved from https://orbit.dtu.dk/en/publications/model-based-monitoring-and-optimization-of-a-bio-based-process

[bit27586-bib-0022] Sin, G. , & Gernaey, K. V. (2016). Data handling and parameter estimation, Experimental methods in wastewater treatment (pp. 201–234). IWA Publishing.

[bit27586-bib-0023] Sin, G. , Ödman, P. , Petersen, N. , Lantz, A. E. , & Gernaey, K. V. (2008). Matrix notation for efficient development of first‐principles models within PAT applications: Integrated modeling of antibiotic production with *Streptomyces coelicolor* . Biotechnology and Bioengineering, 101(1), 153–171. 10.1002/bit.21869 18454503

[bit27586-bib-0024] von Stosch, M. , Davy, S. , Francois, K. , Galvanauskas, V. , Hamelink, J.‐M. , Luebbert, A. , Mayer, M. , Oliveira, R. , O'Kennedy, R. , Rice, P. , & Glassey, J. (2014). Hybrid modeling for quality by design and PAT‐benefits and challenges of applications in biopharmaceutical industry. Biotechnology Journal, 9(6), 719–726. 10.1002/biot.201300385 24806479

[bit27586-bib-0025] Udugama, I. A. , Gargalo, C. L. , Yamashita, Y. , Taube, M. A. , Palazoglu, A. , Young, B. R. , Gernaey, K. V. , Kulahci , Bayer, C. , & M. (2020). The role of Big Data in Industrial (Bio)Chemical Process Operations. Industrial & Engineering Chemistry Research, 59, 15283–15297. 10.1021/acs.iecr.0c01872

[bit27586-bib-0026] Westman, J. O. , Bonander, N. , Taherzadeh, M. J. , & Franzén, C. J. (2014). Improved sugar co‐utilisation by encapsulation of a recombinant *Saccharomyces cerevisiae* strain in alginate‐chitosan capsules. Biotechnology for Biofuels, 7(1), 1–14. 10.1186/1754-6834-7-102 25050138 PMC4094676

[bit27586-bib-0027] Zhou, G. , Jørgensen, J. B. , Duwig, C. , & Huusom, J. K. (2012). State estimation in the automotive SCR DeNOx process IFAC Proceedings Volumes, 45, 501–506. 10.3182/20120710-4-SG-2026.00133We

