# Supplementary material for: Transforming data to information: A parallel hybrid model for real‐time state estimation in lignocellulosic ethanol fermentation
Source: Biotechnol Bioeng. 2020 Oct 15;118(2):579–91. doi: 10.1002/bit.27586 (PMC7894558; doi:10.1002/bit.27586)
Supplement: Supplementary file 1 — Supporting information. [file BIT-118-579-s001.docx]

**Supplementary Material**

## Wheat straw pretreatment and hydrolysis

### I.1.Pretreatment of wheat straw

Five kg of rolled dried wheat straw (provided by TK Energy ApS, Denmark) were steam pretreated at the pilot facilities in the Department of Chemical Engineering at Lund University (Sweden). The 5 kg of straw were soaked in 45 kg of water for an hour (mass ratio 1:10). After one hour, the wet wheat straw was filtered through a 1 mm filter for one hour. Then, the wheat straw was press filtered for 5 min at a pressure of 200 bar, until no more water was extracted from the unit. The remaining water was collected and weighed (11.61 kg). The pressed wheat straw was collected, manually homogenized, and weighed (12.53 kg). Two samples were taken for dry matter analysis, resulting in a dry matter content result of 36.16 % and 37.86 %, respectively. The calculated dry matter content from the weight of the wheat straw was 39.90 %. The pretreatment was done in a steam pretreatment unit, at 200 ºC and 16.8 bar for 10 minutes. Seven runs with 1000 g of pressed wheat straw each were done, generating a final amount of 16.14 kg of pretreated wheat straw. The pretreated wheat straw was collected in 2 containers (each of 1 L of capacity) and stored in the freezer at -20 ºC until use.

### I.2. Enzymatic hydrolysis

The pretreated material (Native dry matter content of 15.4 g/100 g) was pH adjusted to pH 5.0 with 5 M NaOH prior to enzymatic saccharification (the amount of NaOH added was negligible in relation to DM content). One kg of material was transferred to a 2 L laboratory plastic bottle (low-density polyethylene (Kautex Textron, Bonn, Germany)). Five mL of Cellic® CTec2 (Novozymes, Bagsværd, Denmark) were added, corresponding to an enzyme concentration of 6 g Cellic® CTec2 /100 g cellulose (assuming 45 % cellulose in pretreated material). The bottles were carefully sealed and incubated at 50 °C in a rotary drum incubator (Termaks, Bergen, Norway) at 10 rpm for 72 hours, enabling free-fall mixing of the biomass contained in the bottles. This procedure was repeated 15 times to produce a total of 15 L of enzymatically hydrolyzed wheat straw. Prior to further processing, the samples were mixed and homogenized. The samples were stored at -20 °C to avoid microbial spoilage.

## Reaction rates.

*Table S1. Processes used in the kinetic model.*

| Processes description | Reaction nr |
| --- | --- |
| Glucose uptake | 1 |
| Xylose uptake | 2 |
| Furfural uptake | 3 |
| Furfural 🡪 Furfuryl alcohol | 4 |
| Furfuryl alcohol inhibits glucose uptake | 5 |
| Furfuryl alcohol inhibits xylose uptake | 6 |
| Furfural inhibits glucose uptake | 7 |
| Furfural inhibits xylose uptake | 8 |
| Furfural inhibits 5-HMF uptake | 9 |
| 5-HMF inhibits glucose uptake | 10 |
| 5-HMF inhibits xylose uptake | 11 |
| 5-HMF uptake | 12 |
| 5-HMF 🡪 Acetate | 13 |
| Acetate uptake | 14 |
| Acetate inhibits glucose uptake | 15 |
| Acetate inhibits xylose uptake | 16 |
| Ethanol production | 17 |
| Ethanol inhibits glucose uptake | 18 |
| Ethanol inhibits xylose uptake | 19 |
| Cell growth | 20 |
| Competitive inhibition | 21 |

*Table S2. Reaction rates used in the kinetic model.*

| **Compound** | **Reaction rate** | **Eq. Nr.** |
| --- | --- | --- |
| **Glucose** | $v_{Glu}=-X\cdot\frac{v_{max,Glu}\cdot Glu}{K_{S,Glu}+Glu+\frac{{Glu}^{2}}{K_{i,Glu}}}\cdot\left( 1-\left( \frac{EtOH}{P_{max,Glu}} \right)^{\gamma_{Glu}} \right)\cdot\left( \frac{1}{1+\frac{Fur}{K_{i,Fur,Glu}}} \right)\cdot\left( \frac{1}{1+\frac{FA}{K_{i,FA,Glu}}} \right)\cdot\left( \frac{1}{1+\frac{HMF}{K_{i,HMF,Glu}}} \right)\cdot\left( \frac{1}{1+\frac{HAc}{K_{i,HAc,Glu}}} \right)$ | **(A.1)** |
| **Xylose** | $v_{Xyl}=-X\cdot\frac{v_{max,Xyl}\cdot Xyl}{K_{S,Xyl}+Xyl+\frac{{Xyl}^{2}}{K_{i,Xyl}}}\cdot\left( 1-\left( \frac{EtOH}{P_{max,Xyl}} \right)^{\gamma_{Xyl}} \right)\cdot\left( \frac{1}{1+\frac{Fur}{K_{i,Fur,Xyl}}} \right)\cdot\left( \frac{1}{1+\frac{FA}{K_{i,FA,Xyl}}} \right)\cdot\left( \frac{1}{1+\frac{HMF}{K_{i,HMF,Xyl}}} \right)\cdot\left( \frac{1}{1+\frac{HAc}{K_{i,HAc,Xyl}}} \right)\cdot\left( \frac{1}{1+\frac{Glu}{K_{i,Glu,Xyl}}} \right)$ | **(A.2)** |
| **Furfural** | $v_{Fur}=-X\cdot\frac{v_{max,Fur}\cdot Fur}{K_{SP,Fur}+Fur}$ | **(A.3)** |
| **5-HMF** | $v_{HMF}= -X\cdot\frac{v_{max,HMF}\cdot HMF}{K_{SP,HMF}+HMF}\cdot\left( \frac{1}{1+\frac{Fur}{K_{i,Fur,HMF}}} \right)$ | **(A.4)** |
| **Acetic acid** | $v_{HAc}= -X\cdot\frac{v_{max,HAc}\cdot HAc}{K_{SP,HAc}+HAc}+ Y_{{HAc}/{HMF}}\cdot v_{HMF}$ | **(A.5)** |
| **Furfuryl alcohol** | $v_{FA}= -Y_{{FA}/{Fur}}\cdot v_{Fur}$ | **(A.6)** |
| **Ethanol** | $v_{EtOH}= -Y_{{EtOH}/{Glu}}\cdot v_{Glu}-Y_{{EtOH}/{Xyl}}\cdot v_{Xyl}$ | **(A.7)** |
| **Biomass** | $v_{X}= -Y_{X/{Glu}}\cdot v_{Glu}-Y_{X/{Xyl}}\cdot v_{Xyl}$ | **(A.8)** |

List of parameters.

*Table S3. List of parameters used in the kinetic model*

| **Parameter** | **Description** | **Reference** | **Initial guess** | **Value after PE** | **Uncertainty** | **Unit** |
| --- | --- | --- | --- | --- | --- | --- |
| ***v_max,Glu_*** | Max biomass specific glucose uptake rate | [17] | 1.001 | 1.927 | 1.83E-5 | g Glu (g X)^-1^ h^-1^ |
| ***K_S,Glu_*** | Affinity constant glucose | [17] | 0.565 | 0.565 | - | g/L |
| ***K_iP,Glu_*** | Glucose substrate inhibition constant | [17] | 4890 | 4890 | - | g/L |
| ***v_max,Xyl_*** | Max biomass specific xylose uptake rate | [17] | 0.210 | 1.622 | 1.62E-2 | g Xyl (g X)^-1^ h^-1^ |
| ***K_S,Xyl_*** | Affinity constant xylose | [17] | 3.400 | 3.400 | - | g/L |
| ***K_iP,Xyl_*** | Xylose substrate inhibition constant | [17] | 18.100 | 18.100 | - | g/L |
| ***v_max,Fur_*** | Max biomass specific furfural uptake rate | [18] | 0.168 | 0.168 | - | g Fur (g X)^-1^ h^-1^ |
| ***K_S,Fur_*** | Affinity constant furfural | [18] | 0.050 | 0.050 | - | g/L |
| ***Y_FA/Fur_*** | Yield FA/Fur | [18] | 1.020 | 1.020 | - | g/g |
| ***K_i,FA,Glu_*** | Inhibition constant FA on glucose | [18] | 5.000 | 5.000 | - | g/L |
| ***K_i,FA,Xyl_*** | Inhibition constant FA on xylose | [18] | 6.000 | 6.000 | - | g/L |
| ***K_i,Fur,Glu_*** | Inhibition constant Fur on glucose | [18] | 0.750 | 0.750 | - | g/L |
| ***K_i,Fur,Xyl_*** | Inhibition constant Fur on xylose | [18] | 0.350 | 0.350 | - | g/L |
| ***K_i,Fur,HMF_*** | Inhibition constant Fur on HMF | [18] | 0.250 | 0.250 | - | g/L |
| ***K_i,HMF,Glu_*** | Inhibition constant HMF on glucose | [18] | 2.000 | 2.000 | - | g/L |
| ***K_i,HMF,Xyl_*** | Inhibition constant HMF on xylose | [18] | 10.000 | 10.000 | - | g/L |
| ***v_max,HMF_*** | Max biomass specific HMF uptake rate | [18] | 0.315 | 0.315 | - | g HMF (g X)^-1^ h^-1^ |
| ***K_S,HMF_*** | Affinity constant HMF | [18] | 0.500 | 0.500 | - | g/L |
| ***v_max,HAc_*** | Max biomass specific HAc uptake rate | [18] | 0.044 | 4.4E-5 | - | g HAc (g X)^-1^ h^-1^ |
| ***K_S,HAc_*** | Affinity constant HAc | [18] | 2.500 | 2.500 | - | g/L |
| ***Y_HMF/HAc_*** | Yield HMF/HAc | [18] | 0.534 | 0.534 | - | g/g |
| ***K_i,HAc,Glu_*** | Inhibition constant HAc on glucose | [18] | 3.740 | 5.670 | 2.00E-3 | g/L |
| ***K_i,HAc,Xyl_*** | Inhibition constant HAc on xylose | [18] | 3.750 | 3.750 | - | g/L |
| ***Y_EtOH/Glu_*** | Yield EtOH/Glu | [17] | 0.420 | 0.420 | - | g/g |
| ***Y_EtOH,Xyl_*** | Yield EtOH/Xyl | [17] | 0.240 | 0.240 | - | g/g |
| ***P_max,Glu_*** | Inhibition constant EtOH on glucose | [17] | 103.000 | 103.000 | - | g/L |
| ***γ_Glu_*** | Exponential factor inhibition EtOH on glucose | [17] | 1.420 | 1.420 | - | - |
| ***P_max,Xyl_*** | Inhibition constant EtOH on xylose | [17] | 60.200 | 60.200 | - | g/L |
| ***γ_Xyl_*** | Exponential factor inhibition EtOH on xylose | [17] | 0.608 | 0.608 | - | - |
| ***Y_X/Glu_*** | Yield X/Glu | [17] | 0.115 | 0.115 | - | g/g |
| ***Y_X/Xyl_*** | Yield X/Xyl | [17] | 0.162 | 0.162 | - | g/g |
| ***K_i,Glu,Xyl_*** | Inhibition constant Glu on xylose | Estimated | 8.000 | 13.763 | 8.80E-4 | g/L |

## Sensitivity and identifiability analysis

## *IV.1. Sensitivity functions.*

The significance of each parameter was calculated based on their sensitivity functions on the model outputs. The sensitivity functions, s_i,j_, are defined as shown in **Equation A.6**.

$s_{i,j}= \left| \frac{\partial\eta_{j}\left( \theta_{i} \right)}{{\partial\theta}_{i}} \right|_{\theta_{i}}$ **(A.6)**

where ${\partial\eta_{j}\left( \theta_{i} \right)}/{{\partial\theta}_{i}}$ represents the change in the output *η_j_* due to a change in the parameter *θ_i_*. The parameters were then ranked based to their significance on the different outputs as shown in **Equation A.7**.

$\delta^{msqr}= \sqrt{\frac{1}{n}\sum_{j}^{n} s_{i,j}^{2}}$ **(A.7)**

A detailed explanation of the sensitivity and identifiability analysis is provided by Sin *et al.*  [20]. The local sensitivity analysis (**Figure A.1)** revealed that only 7 parameters had a significant effect on the output (*v_max,Glu_, v_max,Xyl_, K_iP,Xyl_, K_iHAc,Glu_, K_iHAc,Xyl_, P_max,Xyl_ and γ_Xyl_*).

## *IV.II. Identifiability analysis.*

The identifiability analysis of all the possible combinations of parameters was performed to identify non-linearly correlated subsets of parameters. A collinearity index was calculated by taking the inverse of the smallest eigenvalue of the normalized sensitivity matrix for each given subset of parameters. A detailed description of the identifiability analysis method is given by Brun *et al,* [19]. A subset of parameters with a collinearity index below 15 was considered as approximately non-linearly correlated. The identifiability analysis (**Figure A.2**) showed that a maximum of 12 parameters could be simultaneously identified.

**Figure S.1.** Results of the local sensitivity analysis. The significance of each parameter on the different model outputs is represented in a spider plot.

**Figure S.2.** Results of the local identifiability analysis.

As a result of the sensitivity and identifiability analysis, a subset of three non-linearly correlated and significant parameters (*v_max,Glu_, K_iHAc,Glu_* and *K_i,Glu,Xyl_*) was chosen for the parameter estimation.

## Results of the parameter estimation and validation of the model

**Figure S.3. Parameter estimation and validation of the kinetic model.**

## Synthetic samples used for the calibration of the PLS models.

*Table S4. Concentration of glucose, xylose and ethanol in the calibration set of the PLS models*

| **Sample Nr.** | **Glucose [g/L]** | **Xylose [g/L]** | | **Ethanol [g/L]*** |
| --- | --- | --- | --- | --- |
| 0 | 2.00 | 1.25 | 14.50 | |
| 1 | 0.00 | 17.50 | 10.00 | |
| 2 | 34.00 | 5.00 | 23.50 | |
| 3 | 38.00 | 21.25 | 31.00 | |
| 4 | 36.00 | 16.25 | 11.50 | |
| 5 | 24.00 | 6.25 | 20.50 | |
| 6 | 18.00 | 15.00 | 19.00 | |
| 7 | 12.00 | 25.00 | 16.00 | |
| 8 | 26.00 | 2.50 | 25.00 | |
| 9 | 22.00 | 12.50 | 12.00 | |
| 10 | 10.00 | 10.00 | 8.50 | |
| 11 | 8.00 | 23.75 | 32.50 | |
| 12 | 4.00 | 3.75 | 26.50 | |
| 13 | 30.00 | 7.50 | 7.00 | |
| 14 | 32.00 | 0.00 | 2.50 | |
| 15 | 20.00 | 18.75 | 13.00 | |
| 16 | 28.00 | 13.75 | 28.00 | |
| 17 | 40.00 | 22.50 | 5.50 | |
| 18 | 14.00 | 11.25 | 17.50 | |
| 19 | 16.00 | 8.75 | 29.50 | |
| 20 | 6.00 | 20.00 | 4.00 | |

* Note that the lowest concentration of ethanol left after stripping was 2.5 g/L.

## PLS calibrations

**Figure S.4. Calibration of the PLS models.**
